# Supplementary material for: Metrological Protocols for Reaching Reliable and SI-Traceable Size Results for Multi-Modal and Complexly Shaped Reference Nanoparticles
Source: Nanomaterials (Basel). 2023 Mar 9;13(6):993. doi: 10.3390/nano13060993 (PMC10057439; doi:10.3390/nano13060993)
Supplement: Supplementary file 1 [file nanomaterials-13-00993-s001.zip › nanomaterials-2210115-supplementary.pdf]

nanomaterials

Supplementary Materials

Metrological protocols for reaching reliable and SI-traceable size results for multi-modal and complex-shaped reference nanoparticles

Nicolas Feltin [1,*], Loïc Crouzier [1], Alexandra Delvallée [1], Francesco Pellegrino [2], Valter Maurino [2], Dorota Bartczak [3], Heidi Goenaga-Infante [3], Olivier Taché [4], Sylvie Marguet [4], Fabienne Testard [4], Sébastien Artous [5], François Saint-Antonin [5], Christoph Salzmann [6], Jérôme Deumer [7], Christian Gollwitzer [7], Richard Koops[8], Noham Sebaïhi[9], Richard Fontanges[10], Matthias Neuwirth[11], Detlef Bergmann[11], Dorothee Hüser[11], Tobias Klein[11] and Vasile-Dan Hodoroaba [6].

1. Laboratoire National de Métrologie et d'Essais (LNE), 29 Avenue Roger Hennequin, 78197 Trappes, France
2. Dipartimento di Chimica and NIS Inter-Department Centre, University of Torino, Via P. Giuria, 10125 Torino, Italy
3. National Measurement Laboratory, LGC Limited, Queens Road, Teddington TW11 0LY, UK
4. CEA, CNRS, NIMBE, Université Paris-Saclay, 91191 Gif-sur-Yvette, France
5. CEA, Liten, DTNM, Université Grenoble Alpes, 38000 Grenoble, France
6. Federal Institute for Materials Research and Testing (BAM), Unter den Eichen 44-46, 12203 Berlin, Germany
7. Physikalisch-Technische Bundesanstalt (PTB), Abbestraße 2–12, 10587 Berlin, Germany
8. VSL National Metrology Institute, Thjsseweg 11, 2629 JA Delft, The Netherlands
9. National Standards (SMD), FPS Economy, 16 Bd du Roi Albert II, B-1000 Brussels, Belgium
10. Pollen Metrology, 122 Rue du Rocher de Lorzier, Novespace A, 38430 Moirans, France
11. Physikalisch-Technische Bundesanstalt (PTB), Bundesallee 100, 38116 Braunschweig, Germany

* Correspondence: nicolas.feltin@lne.fr

nPSize01 / bimodal gold:

Citrate stabilised colloidal gold NP suspensions with diameter of approximately 30 nm and 60 nm and dispersed in water were purchased from a commercial supplier, BBI solutions Ltd. (UK). The two suspensions were further processed as receive, by mixing at ~ 1:1 ratio (particle number concentration based), ampouled using 5 mL amber glass vials and packaged at LGC (Lucken-walde, Germany). The vials were sterilised using Co^{60} gamma-irradiation with a minimum dose of 35 kGy. Homogeneity and stability in terms of the number concentration of the two size frac-tions were tested with spICP-MS (frequency method) by LGC NMI (Teddington, UK) to ensure the material meets the set requirements and remains stable throughout its shelf-life. The value of the number concentration of the colloid gold particles was determined by LGC (Teddington, UK) with spICP-MS (frequency method) [1] and was (1.88 ± 0.24) $.10^{13}$ kg-1 and (1.93 ± 0.24)$.10^{13}$ kg-1 for the 30 nm and 60 nm fractions, respectively.

nPSize12/ bimodal silica:

nPSize12/ bimodal silica: two batches of colloidal silica NPs suspension in water were synthesized by CEA following a well-known approach [2] with nominal particle diameters of either 30 nm or 60 nm, PSD (Particle Size Distribution) varying from <10% to ≈20%. The concentration (number of particles per ml) for each batches of 30 nm (3.34.10e12 nps/ml) and 60 nm (4.02.10e12 nps/ml) were measured using the CEA laboratory SAXS instrument (Xeuss 2) and protocol described in ref. 3. nPSize12 was obtained following gentle mixing of the suspensions to obtain a ratio of 90% of 30 nm and 10 % of 60 nm. At each steps of the ampouling process, storage and during the characterization campaign,

the stability of the samples was monitored by regularly measuring the sizes, PSD, concentration and ratios with the same SAXS protocol.

nPSize03 / bipyramidal titania:

TiO_2 anatase bipyramids with high percentage of {101} facets exposed were synthesized by hydrothermal treatment of Ti(IV)-triethanolamine complex aqueous solution at 493 K, as described in detail in the literature [4-5]. Briefly, the precursor used for the hydrothermal synthesis is a complex of Ti(IV) with triethanolamine with molar ratio 1:2 ([Ti(TeoaH)2]). The synthesis of the TiO_2 nanoparticles was carried at the following conditions:

- Precursor concentration: 65 mM;
- Triethanolamine: 40 mM;
- Initial pH: 10;
- Temperature: 220°C;
- Time: 50 hours

In general, after dissolution of the required mass of [Ti(TeoaH)2] and of the triethanolamine in MilliQ ultrapure water, pH is adjusted with 0.1 M NaOH, as required. The solution is N2 purged for at least 5 minutes in order to eliminate O_2 before sealing the hydrothermal reactor. The autoclave is heated to 40 °C for 30 minutes, then to the set temperature for the treatment (±1°C) with a ramp of 1 °C min-1. The final temperature is kept constant for 50 hours. The reactor is then cooled in air.

nPSize04 / Gold nanocubes:

Mono-crystalline Au nanocubes were prepared by colloidal chemistry, according to a two-step approach, composed of a seeding process and a growth process [6]. The seed solution was prepared by adding an ice-cold $NaBH_4$ solution (0.42 ml, 10mM) into an aqueous solution composed of a mixture of $HAuCl_4$ (92µl, 10mM) and CTAB (7ml, 100mM). The growth solution was prepared by the successive addition of three aqueous solutions i.e. CTAB (9ml, 22mM), $HAuCl_4$ (0.250ml, 10mM) and ascorbic acid (3ml, 38mM). Next, 20µl of a water-diluted seed solution (1:50) was injected into the growth solution. Purified solutions of nanocubes were obtained by centrifugation twice with water redispersion.

Cetyltrimethylammonium bromide (CTAB ≥ 98%), chloroauric acid ($HAuCl_4{\cdot}3H_2O$), sodium borohydride ($NaBH_4$,99%), and ascorbic acid (99+%) were purchased from Sigma and used as received. Deionized water is used for all experiments.

nPSize07 / Gold nanorods:

Material: Cethyltrimethylammoniumbromide (CTAB, 99%) was purchased from GBiosciences. Tetrachloroauric(III) acid ($HAuCl_4$, extra pure), silver nitrate ($AgNO_3$, extra pure), sodium borohydride ($NaBH_4$, 98%), L-ascorbic acid were obtained from Sigma without further purification. All the glassware was cleaned with aqua regia and washed with distilled water before conducting the experiments.

Protocol: The synthesis if gold nanorods (GNRs) follows a seeded growth protocol in water with first, the synthesis of preformed seeds and secondly, their growth in a growth solution [7]. 300µL of cold $NaBH_4$ (0.01M) was added to a solution made of CTAB (4.6 mM, 0.1M) and $HAuCl_4$ (25µL, 0.05 M) water solutions at 30°C. The growth solution contains CTAB (0.1M), $HAuCl_4$ (0.5 mM), $AgNO_3$ (0.06 mM) and ascorbic acid (0.75 mM) as a mild reducing agent. 120 µl of the CTAB capped seeds suspension was added in the growth solution. The final solution was kept for 2 h at 30°C, then the GNRs were purified by centrifugation (3500 rpm, r =4.5 cm, 20 min) twice with water redispersion.

References:

1. Standard ISO/TS 19590 Nanotechnologies – Size distribution and concentration of inorganic nanoparticles in aqueous media via single particle inductively coupled plasma mass spectrometry, **2017**.
2. Stöber, W.; Fink, A. and Bohn, E. Controlled growth of monodisperse silica spheres in the micron size range. *Journal of colloid and interface science* **1968**, 26(1), 62-69.
3. Geertsen, V.; Barruet, E.; Gobeaux, F.; Lacour, J-L. and Taché O., Contribution to Accurate Spherical Gold Nanoparticle Size Determination by Single-Particle Inductively Coupled Mass Spectrometry: A Comparison with Small-Angle X-ray *Scattering Analytical Chemistry* **2018,** 90 (16), 9742-9750
4. Lavric, V.; Isopescu, R.; Maurino, V.; Pellegrino, F.; Pellutiè, L.; Ortel, E.; Hodoroaba, V.-D. New Model for Nano-TiO_2 Crystal Birth and Growth in Hydrothermal Treatment Using an Oriented Attachment Approach. *Crystal Growth and Design* **2017**, 17 (11), 5640-5651; DOI 10.1021/acs.cgd.7b00302
5. Pellegrino, F.; Raluca, I.; Pellutiè, L.; Sordello, F.; Rossi, A.M.; Ortel, E.; Martra, G.; Hodoroaba, V.-D.; Maurino, V.; Machine learning approach for elucidating and predicting the role of synthesis parameters on the shape and size of TiO_2 nanoparticles, *Scientific Reports* **2020**, 10(11), 18910 . Doi 10.1038/s41598-020-75967-w.
6. Haggui, M.; Dridi, M.; Plain, J.; Marguet, S.; Perez, H.; Schatz, G. C., Wiederrecht, G. P.; Gray, S. K.; Bachelot, R.; Spatial Confinement of Electromagnetic Hot and Cold Spots in Gold Nanocubes. *ACS Nano*, **2012**, 6(2), p1299-1307
7. Gómez-Graña, S.; Hubert, F.; Testard, F.; Guerrero-Martínez, A.; Grillo, I.; Liz-Marzán, L. M. & Spalla, O. Surfactant (bi) layers on gold Nanorods, *Langmuir*, **2012**, 28(2):1453-9. https://doi.org/10.1021/la203451p.
